# A holobiont birth narrative: the epigenetic transmission of the human microbiome

**DOI:** 10.3389/fgene.2014.00282

**Published:** 2014-08-19

**Authors:** Scott F. Gilbert

**Affiliations:** ^1^Department of Biology, Swarthmore CollegeSwarthmore, PA, USA; ^2^Biotechnology Institute, University of HelsinkiHelsinki, Finland

**Keywords:** holobiont, birth, symbiosis, colonization, individuality

## Abstract

This essay plans to explore, expand, and re-tell the human birth narrative. Usually, human birth narratives focus on the origins of a new individual, focusing on the mother and fetus. This essay discusses birth as the origin of a new community. For not only is the eukaryotic body being reproduced, but so also are the bodies of its symbiotic microbes and so is the set of relationships between these organic components. Several parts of the new narrative are surprising: (1) bacterial symbionts might cause some of the characteristics of pregnancy and prepare a symbiotic community for transfer; (2) the first bacterial colonizers of the mammalian organism my enter the fetus prior to the lysing of the amniotic membrane and birth; (3) the same signals that often cause immunological attack against a microbe may serve under these conditions to signal homeostatic stability between symbiont and host; and (4) the mother may actively provide substances that promote the growth and settlement of helpful bacteria. The birth of the holobiont exemplifies principles of co-evolution, co-development, niche construction, and scaffolding. Birth is nothing less than the passage from one set of symbiotic relationships to another.

## Rethinking the birth narrative

This essay plans to explore, expand, and re-tell the human birth narrative. Usually, human birth narratives focus on the origins of a new *individual*, focusing on the heroic trevails of the mother or the amazing journey of fetus. I wish to discuss birth as the origin of a new *community*^1^. For not only is the eukaryotic body being reproduced, but so also are the bodies of its symbiotic microbes and so is the set of relationships between these organic components. Not only are the nuclear and mitochondrial genomes being transmitted, so are the genomes of the symbiotic community, whose microbial genes outnumber those of the eukaryotic component by over 100-fold (Human Microbiome Project Consortium, 2012; McFall-Ngai et al., 2013). Birth is nothing less than the passage from one set of symbiotic relationships to another. The holobionts (mother, fetus, infant) are preserved, but the components of these consortia have changed.

Mammalian birth is one of the great biological stories—the fundamental mammalian symbiosis of mother-and-fetus becoming another fundamental mammalian symbiosis, that of mother-and-child or mother-and-pup. In the classical story, the two partners are obviously the mother and the conceptus (zygote, embryo, fetus, child). Their interactions constitute the grand idea behind the obstetric subspecialty of Maternal-Fetal Medicine, which justifies its existence through the claim that the mother and fetus constitute a co-organized interacting whole, the “maternal-fetal unit.” Indeed, they are a symbiotic unit, a system, where the treatment of one affects the physiology of the other. The mother influences the fetus, providing it with nutrition, oxygen, antibodies, and hormones for its growth. The fetus reciprocally influences the mother, changing her blood circulation, immune responsiveness, and metabolism, while providing her with hormones to retain pregnancy. The physiology of the mother changes as the pregnancy continues, with both the mother and the fetus producing hormones and other growth factors to influence the other's survival and development.

But there is a third major player in this symbiotic mix: the mother's microbes. The pregnant mammal is herself a symbiotic community, a holobiont, composed of numerous species, most of them bacterial (Rosenberg et al., 2007; Gilbert et al., 2012). Nine out of every 10 of the mother's cells are microbial (Bäckhed et al., 2005; Ley et al., 2006) and metagenomic sequencing has shown that each human has entered into partnerships with over 150 species of bacteria (Qin et al., 2010). These bacteria are actively metabolizing nutrients, and the blood being received by the fetus has been substantially altered by the mother's microbes. About 30–35% of the metabolites in mammalian blood has a bacterial origin (Wikoff et al., 2009; McFall-Ngai et al., 2013). Different microbes metabolize dietary products differently, and different diets promote the population of different microbial communities within the mother (Turnbaugh et al., 2006, 2008; Frankenfeld et al., 2014). In mice, for instance, nearly all the blood-born serotonin is made by symbiotic bacteria (Wikoff et al., 2009). So the fetus is not free of a mother's symbiotic associations, even if it is thought that the fetus is sterile or persists in a sterile environment. Rather, the microbes of the mother are interacting with it.

So the fetus survives and develops in a network of symbiotic relationships provided by the pregnant mammal. When the infant/pup is born, these young animals will be going from one set of symbiotic associations to another. They will be leaving the symbiotic consortium of the mother and forming their own symbiotic consortium. They will not be “independent organisms,” nor will they ever be. One is always a holobiont. But at birth, one has to pass through different symbiotic associations. Birth is the process of leaving of one symbiotic association system and forming another.

And remarkably, this transition and transmission is mediated by the maternal holobiont. This transition is not as stark as might be imagined. Rather, the mother is both actively and passively engaged in providing the symbiotic community that will persist over the life of the newborn. The standard scientific birth narrative has been that the fetus is growing in sterile conditions within the amnion, and that bacterial colonization of the fetus is made possible as soon as the amnion breaks and the fetus travels through the birth canal (see Funkhouser and Bordenstein, 2013 for a history and critique of this view). Then, according to this hypothesis, as the fetus traverses the cervix and vagina, the bacteria residing there can enter this new body, whose immune system is not sufficiently mature to attack them. Somehow, though, only certain bacteria can be let into this body, while other bacteria must be kept out. Moreover, only certain areas (gut, pores, mouth, etc.) will be allowed to retain persistent colonies, and these places will select for different types of bacteria.

I wish to critique this standard model more thoroughly, for recent studies find several more interactive and important roles for the mother. The symbiotic microbiome must be understood as constituting a third set of inherited genes. In addition to the nucleus and mitochondria, the symbiotic microbiome is passed from one generation to the next (see Moran, 2007; Douglas, 2010; Gilbert, 2011). This inheritance can be vertically in the germ line (as is often the case in invertebrates) or horizontally by infection (as is often seen in vertebrates). In some cases, both types of transmission are used. In mammals, where the germline is not seen to contain symbiotic bacteria, the newborn acquires its symbionts from its immediate environment. However, the mammalian fetus does not just leave the uterus and passively acquire a new set of symbionts. Rather, the mother actively passes the symbiotic baton to the developing fetus, and she doesn't relinquish control as rapidly and immediately as one might expect from the standard story. Indeed, the colonization of the body, along with the first breath that changes the circulatory system of the newborn, is possibly the most important biological aspect of birth, and the mother will be playing an active role in this process.

## Preparations for delivery

The pregnant mother (i.e., the maternal holobiont) changes dramatically during pregnancy, as the body undergoes hormonal, immunological, and metabolic changes. These include fat gain starting early in pregnancy, and insulin resistance later in pregnancy. These two metabolic conditions, which are often detrimental to men and to non-pregnant women, are thought to be beneficial during pregnancy. At this time, increased adiposity and increased insulin resistance are thought to support fetal growth and to prepare the mother for lactation (Di Cianni et al., 2003; Lain and Catalano, 2007; Nelson et al., 2010). As we will see, this preparation for lactation is critical for the handover of symbiotic community from the mother to the infant.

Employing 91 women and assuming that stool samples accurately reflect the intestinal microbiota, Koren et al. (2012) used polymerase chain reactions to show that the gut microbiome of pregnant women changed dramatically during pregnancy. These included women who took probiotics during pregnancy and who had used antibiotics during either the first or second trimester. In most of the women, the first-trimester gut bacteria were similar to that of the general non-pregnant population, but the third- trimester samples differed significantly. In a majority of women, the relative abundances of Proteobacteria and Actinobacteria increased substantially (*P* = 0.0004 and 0.003, respectively) during this time between 13 and 33 weeks of pregnancy. Moreover, the microbial community became more streamlined, with the diversity much reduced by the third trimester.

The importance of these bacteria to normal pregnancy was demonstrated by transferring the bacteria from the healthy first-trimester and third-trimester women into healthy female germ-free mice. The mice receiving the bacteria from the stools of first-trimester pregnant women remained normal. However, within 2 weeks, the healthy formerly germ-free mice that received the third-trimester bacteria had a pregnancy-like metabolic syndrome, complete with insulin insensitivity, excessive weight gain, and increased markers of inflammation.

These bacteria were derived from the gut. The vaginal bacterial community has also been analyzed (Aagaard et al., 2012; Romero et al., 2014) and was found to have a dynamic pattern during gestation, returning to an essentially non-pregnant state toward the end of pregnancy. *Lactobacillus* species, however, appear to be enriched during pregnancy. Several enriched Lactobacci species digest glycogen, and they produce an acidic environment that prevents pathogenic infection (O'Hanlon et al., 2011). One particular Lactobacillus, *L. johnsonii* is found in the gut and vagina. In the gut it is an important component of the upper digestive tract and is critical for the processing of bile salts (Pridmore et al., 2004). However, *L. johnsonii* also produces a bacteriotoxic compound, Lactacin F, which prevents the growth of particular bacterial pathogens (Abee et al., 1994). So the vaginal microbiota appear to be helping the mother ward off infections of the reproductive tract during pregnancy.

Thus, there is a dramatic remodeling of the gut and vaginal microbiological communities over the course of pregnancy. Although the mechanisms have not been delineated, it appears that the hormonal changes of the host are changing the population of microbes in the gut and vagina. These are the microbial populations that will be experienced by the late-stage fetus as it leaves the birth canal.

## Initial colonization: the sooners and the initiation of labor

It has long been assumed (Tissier, 1900) that the fetus develops within a sterile environment, and that when the amnion bursts during labor, the colonization could begin. The first microbes would reach the fetus as it was being born. These would be the resident microbes of the birth canal. Later, microbes from the mother's breast and skin would be in line for colonizing the newborn baby.

This would be the obvious way. However, there appear to be “sooners.”^2^ New evidence affirms that the first settlers of this newfound fetal territory are colonists from the mother's microbiome that gain entry into the developing fetus, bypassing the placental and amniotic barriers. Strangely, bacteria have been found in normal amniotic fluid, in the umbilical cord, and in infant's first bowel movements, the meconium (see Funkhouser and Bordenstein, 2013). This would indicate that the bacteria were already in the fetus before birth. To experimentally observe whether maternal bacteria could be transferred into the fetus, Jimenez et al. (2008) fed pregnant mice milk that had been inoculated with genetically labeled *Enterococcus faecum* bacteria. A day before the mice were to be naturally born, the researchers performed a Caesarean section on the mice, delivering them aseptically. They found that their first bowel movement not only contained bacteria, but that some of the bacteria had the transgenic label that could only have been received through the oral cavity or gut of their mothers.

However, while the vaginal microbiota might prevent pathogenic infection, and while the gut bacteria may induce a metabolic syndrome in the mother, these sources do not appear to be where the initial colonizers are coming from. Surprisingly, data suggest that the first colonizing bacteria arise from the mouth and then work their way into the fetus while it is still within the amnion. Molecular studies also indicated that the early colonization of human neonates appears to be accomplished by bacteria originating from the oral cavity (Palmer et al., 2007; Human Microbiome Project Consortium, 2012; Jost et al., 2012; Milisavljevic et al., 2013). According to sequencing data (Stout et al., 2013; Aagaard et al., 2014; Prince et al., 2014), the neonatal gut microbiota do not resemble the maternal vaginal or gut microbiota, but contain populations of bacteria derived from a placental source that stems from the oral cavity. Moreover, specific bacteria that are found normally or pathologically in the oral cavity (and not in the lower gut or vagina) have been isolated from human amniotic fluid (Ernest and Wasilauskas, 1985; Douvier et al., 1999; Bearfield, 2002; and Han et al., 2004). Bacteria were formerly thought to be found in placentae only in those mothers at risk for preterm labor. However, Stout et al. (2013) have questioned this idea by identifying intracellular bacteria in normal term and preterm placentae^3^.

The mechanism by which oral bacteria can get to the placenta is not yet known. However, one possibility is that *dendritic cells* of the oral cavity transport bacteria to lymphatic tissue in the placenta (see Donnet-Hughes et al., 2010; Funkhouser and Bordenstein, 2013). The oral mucosa contains numerous populations of dendritic cells, and these cells migrate through the blood and lymphatic vesicles to lymphoid tissues to mediate tolerance or immunogenicity (Hovav, 2014). When reaching the lymphoid tissues, the dendritic cells can diapedese across the endothelial cells into the lymphoid tissues (see de la Rosa et al., 2003; Johnson and Jackson, 2014). In many cases, they transport bacteria or other potential pathogens with them, and they present these microbial cells to the lymphocytes. The uterine decidua has a population of resident lymphocytes, and these cells are essential for normal implantation and the lack of rejection of the fetus (Blois et al., 2004; Juretic et al., 2004; Laskarin et al., 2007; Zarnani et al., 2008). So the oral cavity has a mucosa with associated dendritic cells, and the placenta has a lymphoid region capable of receiving dendritic cells. Recently, it was shown that dendritic cells carrying pathogens in them can migrate to the placenta and enable their parasitic passengers to infect the fetus. The transplacental passage of the intracellular *Toxoplasmosis*-like parasite *Neospora caninum* in mice appears to be facilitated by such dendritic cells. Inoculation of pregnant mice with dendritic cells infected with *Neospora* resulted in the migration of these dendritic cells to the placenta, the transmission of the parasite to the offspring, and often the resulting neonatal death (Collantes-Fernandez et al., 2012). There is therefore a pathway by which bacteria in the oral cavity can be transported to the placenta.

But the innate immune system of the fetus should prevent these bacteria from entering the fetal gut. The proteins that regulate immediate (“innate”) immune responses against bacteria in the adult are the Toll-like receptors (TLRs). Interestingly, the activity of these receptors appears to be down-regulated in the fetus. The amniotic fluid, in addition to providing suspension and anti-dessication protection to the embryo, also contains large concentrations of Epidermal Growth Factor. This protein prevents the function of the TLRs. So while the early digestive tract is being bathed by amniotic fluid (the mouth and anus are open and exposed to amniotic fluid), the bacteria might be accepted as colonizers (Good et al., 2012). So there is a pathway through which bacteria in the oral cavity can become the first colonizers of the fetus, even before the amniotic membrane has lysed.

## The second wave: the colonization of the colon

When John Donne famously wrote that “No man is an island,” he was fully correct from the sociological perspective. But from the bacterial perspective, a man is a remarkable island, and the rules of island colonization hold for bacteria (Costello et al., 2012). Those bacteria who arrrive first get a wide choice of options, and they restrict the conditions for the next wave of settlers. Once the amnion has broken, the fetus is exposed to a wide variety of microbes, mostly from the gut and birth canal. Vaginal delivery exposes the fetus and newborn to the microbes of the mother's vagina and gut, a microbiome that has changed over the course of pregnancy (Tannock et al., 1990; Makino et al., 2011). These bacteria appear to be very important, as babies born through Caesarean section (i.e., not passing through the birth canal) have an altered bacterial colonization pattern early in life compared with vaginally delivered babies (Ley et al., 2006; Makino et al., 2013). These gut and vaginal bacteria initiate new host-symbiont relationships, and they will have an important role in the health of the holobiont (Conroy et al., 2009; Le Huërou-Luron et al., 2010). Babies born vaginally have gut bacterial communities that resemble those of the maternal vagina (Matsumiya et al., 2002; Dominguez-Bello et al., 2010). Those babies born by Caesarean section receive many of their colonizers from the hospital environment and from the mother's skin (Martirosian et al., 1995; Dominguez-Bello et al., 2010). Mother's milk also supplies bacteria that will reside in the newborn's gut (Martín et al., 2003; Collado et al., 2009; Solís et al., 2010; Garrido et al., 2012).

It takes over a year for the babies born by C-section to have a similar bacterial profile, and during this time, they have a lower microbial diversity, delayed colonization of important microbes (such as *Bacteroides*) and reduced lymphocyte responses (Guarner and Malagelada, 2003; Jakobsson et al., 2014). The ability of the fetus to decide which bacteria stay and which ones must be excluded is still very much a mystery. The process of colonization appears to involve many of the same molecules that are usually used to attack bacteria. It seems that at the core of either acceptance or rejection is recognition, and the reaction (rejection or tolerance) depends on the context in which the fetus receives these microbes.

Immunology is the science of recognition in contexts. The same signal may call out for destruction or synthesis depending on how it is presented. Here, the host recognizes symbiotic bacteria apparently by the same sets of molecules usually used to attack bacteria; but the turns this recognition into acceptance rather than attack, and in this particular context, these bacteria are actually encouraged to settle into our guts (Chu and Mazmanian, 2013; Lee et al., 2013). This, along with the bacterial regulation of pregnancy weight gain and the entry of bacteria into the fetus prior to tocolysis, has been another surprise. The innate immune system had been thought to recognize bacteria by their pathogen-associated molecular patterns (PAMPs) by their pattern recognition receptors (PRRs). It now turns out that PAMPs are found in all microbes, including symbionts, and the agents recognized by the host's PRRs are now called “microbe-associated molecular patterns” (MAMPs). In mice (as well as in numerous other organisms, including hydra and *Drosophila*), the activation of PRRs in the newborn induces a homeostatic integration of host and symbiont. It appears that the composition of the early microbiome dictates whether the response of the cells is inflammatory or tolerant (See Chu and Mazmanian, 2013).

## The power of positive selection

Now that the newborn's body is being colonized by microbes originating from the gut, vaginal, and oral cavities of the mother, the problem becomes one of specificity: Which bacteria are going to remain and which are to be eliminated? This is a critical question, and one in which research is just beginning. However, some of the research is pointing out how important the mother is in determining which populations persist. One of the most important bacteria present in the mother's intestines is *Bifidobacteria*. These bacteria provide several services to the neonate (Garrido et al., 2012). First, they actively prevent the colonization of the gut by pathogenic bacteria and help induce and sustain the immune system. Second, they provide essential vitamins to the infant (Lievin et al., 2000; Schell et al., 2002; Fukuda et al., 2011). They also increase the tight junctions that are necessary for tightly linking the intestinal epithelial cells together (Chichlowski et al., 2012). Surprisingly, this tightening of intestinal epithelial binding may be essential for the cognitive health of the infant (Hsiao et al., 2013). Bifidobacteria is a good bacterium to have as one of the colonizers of the gut, and genetic evidence supports the idea that mammals and Bifidobacteria have a co-evolutionary history of helping each other for at least 200 million years.

These Bifidobacteria are encountered as the fetus squeezes through the birth canal. Makino et al. (2013) found that specific strains of *Bifidobacteria* become translocated from the mother's intestine to the newborn's gut. When delivered vaginally, monophyletic representatives of the woman's intestinal *Bifidobacteria* formed colonies in the newborn within 3 days of birth. This did not occur in those babies born by Caesarean section, where Bifidobacterial counts were lower after the first week (Makino et al., 2013).

And the mother supports the growth of the newborn's Bifidobacteria colonies. One of the most interesting components of a newborn's diet is mother's milk. And here came another surprise: some of the complex sugars found in human mothers' milk are not digestible by the infant. Rather, they serve as food for certain bacterial symbionts such as *Bifidobacteria* that help the infants' bodies develop (Sela et al., 2011; Zivkovic et al., 2011; Yoshida et al., 2012; Underwood et al., 2013).

The genome of one of the most common Bifidobacteria (*B. longum subspecies infantis*) has been sequenced and shown to contain a remarkable region of DNA—a series of genes linked together and dedicated to the intake and digestion of complex sugars found specifically in the earliest secretions of mother's milk. This small (43 kb) unit is not found in related bacteria that are not part of the gut microbiome, and these data indicate a remarkable co-evolution between this symbiotic bacterium and its host, suggesting that the host product (human milk) and the microbial genome enabling the bacteria to use this product have reciprocally formed each other (Sela et al., 2008). Thus, there would be a co-evolution of human and microbe for the purpose of gut colonization by this microbe, a colonization that would benefit both.

Not only do the sugars in mother's milk feed the “good guys,” these oligosaccharides “prevent the pathogens from latching onto healthy cells, routing trouble-makers into a dirty diaper instead” (Bode, 2012; Manthey et al., 2014; Shugart, 2014). Mother's milk may also help symbiosis by instructing changes in the immune system through microRNAs in its lipid fraction (Munch et al., 2013).

Although each baby starts with a unique bacterial profile, within a year the types and proportions of bacteria have converged to the adult human profile that characterizes the human digestive tract (Palmer et al., 2007). Upon weaning and ingesting solid food, the bacterial population changes again, to the more adult form (Pantoja-Feliciano et al., 2013). Interestingly, the types of bacteria allowed to colonize depend on (1) the prevalence of a particular species of microbes in the environment; (2) which microbes have already entered the gut; (3) the genetics of the digestive tract; and (4) the diet one receives (Nicholson et al., 2012; Pacheco et al., 2012; Kashyap et al., 2013).

## Holobiont procreation

So we have re-told the birth narrative from a holobiont perspective. In doing so, several surprising hypotheses have emerged, ideas that had not been part of the traditional account of pregnancy:

- bacteria help regulate pregnancy
- bacteria can enter the fetus before the amnion breaks
- the activation of PRRs can mediate symbiosis as well as immune attack
- some material in mother's milk is for the bacteria, not the infant.

In this new narrative, we see “birth” as involving the reproduction of the holobiont. In other words, both the mammal and her persistent microbial populations have to be reproduced. Both niche construction and scaffolding are critical. *Niche construction* is defined as “the process whereby organisms, through their metabolism, their activities and their choices, modify their own and/or each other's niches” (Odling-Smee et al., 2003, p. 419). Our symbiotic bacteria employ such niche construction in specifying their environment by changing their host's development. The niches in which bacteria reside are to a large part generated by the bacteria, themselves. Some of the symbiotic microbes in the mouse intestine, for instance, induce gene expression in the gut epithelia not only to help the host, but to help themselves. The normal gut microbes, such as *Bacteroides*, induce gene expression in the Paneth cells of the intestine, instructing these cells to produce two compounds—angiogenin-4 and RegIII—that prevent the colonization of the intestine by *other* species of microbes. *Bacteroides, Escherichia coli*, and other symbiotic species are impervious to this compound, while several pathogenic Gram-positive bacteria (*Enterococcus faecalis* and *Listeria monocytogenes*) are wiped out by it (Hooper et al., 2003; Cash et al., 2006). These bacteria are enemies of Bacteroides and of the mammalian host. Thus, the microbial species is modifying its niche, causing its environment (i.e., the mammal) to change in such a way that they can better survive.

*Scaffolds* are “material environmental inputs with organizations that are sensitive and responsive to the developmental state of the developmental system being scaffolded” (Griesemer, 2014). A scaffold facilitates developmental processes that would be difficult or costly without it, and the scaffold is often temporary. Such scaffolds are critical and reciprocal parts of the holobiont's birth. First, we've seen that the bacteria are part of the scaffolding that allows human reproduction. If some of the “symptoms” of late pregnancy that support the fetus and its delivery are caused by bacteria, then the bacteria is part of the scaffolding of human reproduction. And if the milk sugars of the mother and the modified immune system of the newborn enable the successful reproduction of a particular set of bacteria (that will enable the completion of the developmental capacities of the newborn), then humans are a critical scaffolding for the reproduction of the microbes.

The mother, through her hormones, anatomy, and milk production, is in large part responsible for the successful handing over of the fetus to a new set of symbionts. Going from the maternal environment to the outside world is not merely leaving a symbiotic support system and gaining “independence.” There is no such thing as “independence.” It's mutual dependency all the way down, and birth is the exchanging of one symbiotic system for another.

### Conflict of interest statement

The author declares that the research was conducted in the absence of any commercial or financial relationships that could be construed as a potential conflict of interest.

## Footnotes

^1^Always be wary of male scientists telling birth narratives.

^2^“Sooner” comes from an American slang for those European settlers who entered the Oklahoma Territories of the United States prior to the legal opening of the land.

^3^This has led Prince et al. (2014) to speculate that these bacteria may be involved in the aberrant timing of parturition. It had been thought that preterm births were caused by infections ascending from the vagina. However, recent studies (Madianos et al., 2013) shows that these bacteria are not representative of pathogenic vaginal microbes but are most likely a set of oral microbes. Moreover, periodontal pathogens might reach the fetus and cause preterm births or fetal illness. These, according to Prince and colleagues, would be those that had colonized the placenta.
